# Vitexin Attenuates the Growth and Glycolysis of Acute Myeloid Leukemia Cells by Suppressing the HIF‐1α‐Modulated YAP Pathway Under Hypoxic Conditions

**DOI:** 10.1002/kjm2.70111

**Published:** 2025-09-25

**Authors:** Ping Wang, Jia‐Jia Zhang, Fang Zhang, Xiao‐Jie Qu, Hui Zhang

**Affiliations:** ^1^ Department of Hematopathology People′s Hospital of Chongqing Banan District Chongqing China; ^2^ Clinical Laboratory People′s Hospital of Chongqing Banan District Chongqing China; ^3^ Department of Traditional Chinese Medicine The First Affiliated Hospital of Henan Polytechnic University Jiaozuo China; ^4^ Department of Respiratory Medicine The First Affiliated Hospital of Henan Polytechnic University Jiaozuo China; ^5^ Department of Endocrinology The First Affiliated Hospital of Henan Polytechnic University Jiaozuo China

**Keywords:** acute myeloid leukemia (AML), glycolysis, HIF‐1α, hypoxia, vitexin

## Abstract

Vitexin, an apigenin flavone glycoside, exhibits antitumor activity against various cancers including leukemia. Hypoxia enhances glycolysis, thereby promoting tumor growth. In this study, we aimed to explore the effects of vitexin on hypoxia‐induced growth and glycolysis in acute myeloid leukemia (AML) cells and determine the underlying molecular mechanisms. Our findings showed that vitexin inhibited the hypoxia‐induced increase in viability and proliferation of AML cells. Vitexin suppressed glucose uptake, lactate production, and the expression of hexokinase 2, pyruvate kinase M2, and lactate dehydrogenase A in hypoxia‐exposed AML cells. Vitexin also blocked the hypoxia‐induced increase in hypoxia‐inducible factor 1α (HIF‐1α) and Yes‐associated protein (YAP) expression, as well as the decrease in p‐YAP expression. In addition, our results demonstrate that the YAP pathway is regulated by HIF‐1α in hypoxia‐exposed AML cells and participates in the inhibitory effects of vitexin on hypoxia‐induced AML cell growth and glycolysis. These results indicate that vitexin prevents hypoxia‐induced growth and glycolysis in AML cells by regulating the HIF‐1α/YAP signaling pathway.

## 1 Introduction

Acute myeloid leukemia (AML), a disorder of the bone marrow, is a hematological malignancy of stem cell precursors caused by genetic alterations [1]. Despite improved outcomes in some subgroups, AML continues to have a high mortality rate [2]. Further research into the underlying pathogenic mechanisms is required to achieve remarkable advances in new treatments for the disease.

Hypoxia, a condition of low oxygen levels, contributes to both acute and chronic diseases, including diverse malignancies [3]. Multiple oncogenic pathways are activated under hypoxic conditions, leading to cancer progression [4]. Hypoxia can also induce multiple metabolic processes such as glycolysis, thereby contributing to cancer development [5]. Hexokinase 2 (HK2), pyruvate kinase M2 (PKM2), and lactate dehydrogenase A (LDHA), three key enzymes commonly used as glycolytic markers, are upregulated in response to hypoxia [6]. Hypoxic effects are orchestrated by hypoxia‐inducible factors (HIFs), which mediate the expression of several genes involved in cancer progression [5]. Hypoxia‐inducible factor 1α (HIF‐1α), the principal regulator of hypoxia, promotes tumor cell glycolysis, emerging as a novel therapeutic target to treat hypoxia‐associated diseases [7]. Over the past few decades, the interactions between hypoxia and AML progression have been established [8]. Therefore, controlling hypoxia may prevent AML progression and achieve optimal outcomes in patients with AML.

Yes‐associated protein (YAP) is an oncoprotein encoded by the *YAP* gene, located in chromosome 11q22 genomic region [9]. Activation of YAP is critical for cancer cell growth under hypoxic conditions [10]. YAP phosphorylation inhibits YAP activity by promoting the exclusion of YAP from the nucleus into the cytoplasm [11]. Hypoxia induces YAP activation and accelerates hepatocellular carcinoma cell glycolysis [12]. Under hypoxic conditions, HIF‐1α plays a critical role in promoting YAP expression [13]. Therefore, targeting YAP is a promising strategy for cancer treatment.

**FIGURE 1:**
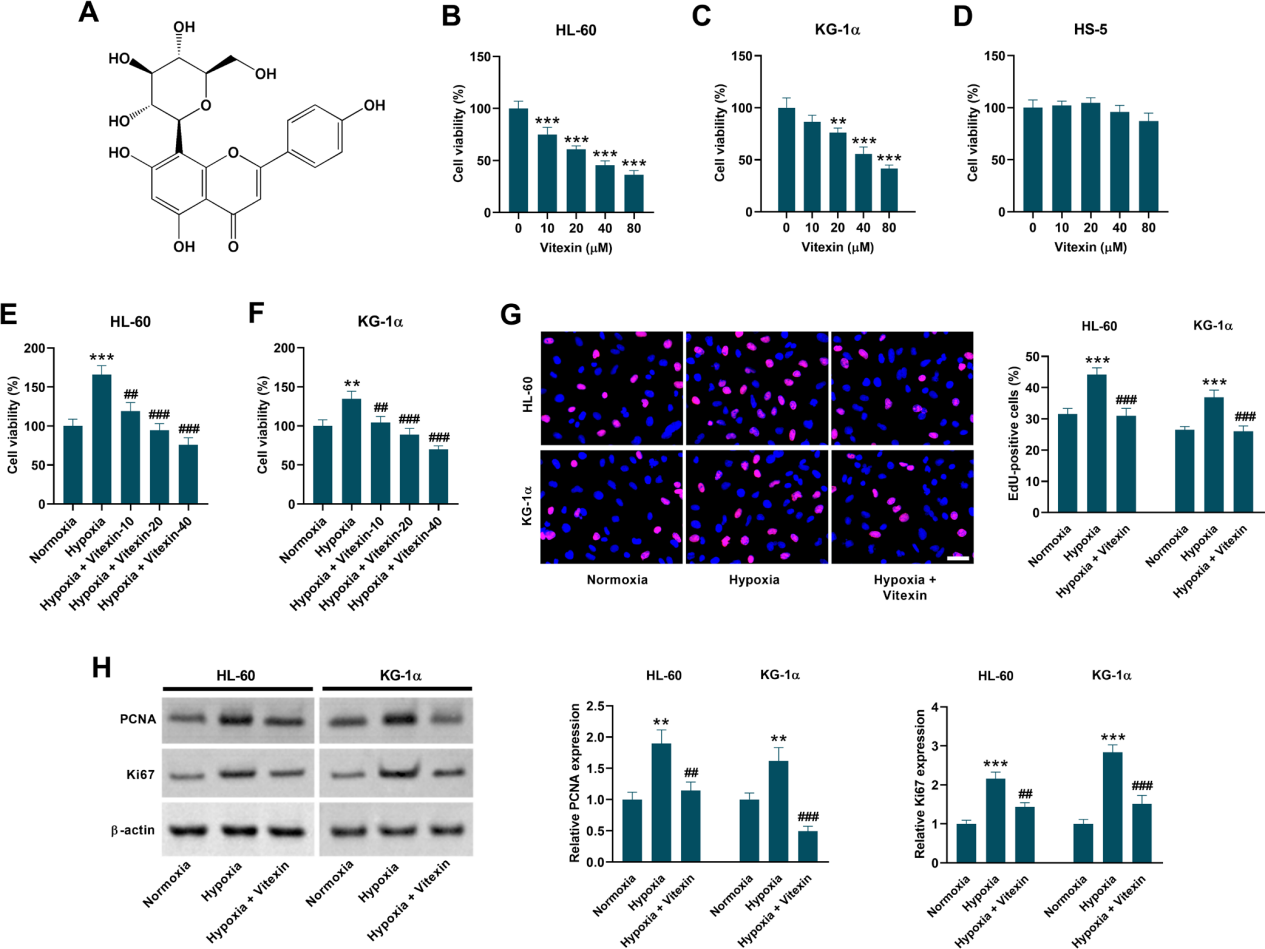
Vitexin inhibits hypoxia‐induced increase in the viability and proliferation of HL‐60 and KG‐1α cells. (A) Chemical structure of vitexin. (B, C) The viabilities of HL‐60 and KG‐1α cells were determined using MTT assay 48 h after treatment with different concentrations of vitexin. Data are presented as mean ± SEM. **p < 0.01, ***p < 0.001 vs. control group. (D) The viability of HS‐5 cells was determined using MTT assay 48 h after treatment with different concentrations of vitexin. Data are presented as mean ± SEM. (E, F) Vitexin treatment suppressed the hypoxia‐induced increase in viability of HL‐60 and KG‐1α cells. Cells were treated with 10, 20, and 40 μM vitexin under hypoxic conditions for 48 h, and cells under normoxic conditions served as the control group. Data are presented as mean ± SEM. **p < 0.01, ***p < 0.001 vs. Normoxia group. ^##^ *p* < 0.01, ^###^ *p* < 0.001 vs. Hypoxia group. (G) EdU incorporation assay showing the percentage of EdU‐positive cells in both HL‐60 and KG‐1α cells treated with or without 40 μM vitexin under hypoxic conditions for 48 h. Scale bar = 20 μm. Cells under normoxic conditions served as the control group. Data are presented as mean ± SEM. ***p < 0.001 vs. Normoxia group. ^###^ *p* < 0.001 vs. Hypoxia group. (H) Western blot analysis showing the expression levels of proliferation‐associated proteins including PCNA and Ki67 in both HL‐60 and KG‐1α cells treated with or without 40 μM vitexin under hypoxic conditions for 48 h. Cells under normoxic conditions served as the control group. Data are presented as mean ± SD. **p < 0.01, ***p < 0.001 vs. Normoxia group. ^##^ *p* < 0.01, ^###^ *p* < 0.001 vs. Hypoxia group.

Vitexin (Figure 1A) is an apigenin flavone glycoside that is currently receiving increasing attention from researchers due to its diverse biological advantages under different disease conditions [14]. Several studies have indicated the potential use of vitexin for the treatment of various cancers, including leukemia [15]. Sarkar et al. reported that vitexin induces apoptosis in leukemia cells by generating free radicals such as ROS and NO, causing cell membrane damage and nuclear condensation [16]. Another study reported that apoptosis in leukemia cells triggered by vitexin is mediated by the mitochondrial signaling pathway [17]. Collectively, these findings suggest that vitexin may serve as a promising therapeutic agent for human leukemia. Although previous studies have shown that vitexin exerts anti‐tumor activity in AML cells under normoxic conditions, whether vitexin affects the hypoxia‐induced malignant properties of AML cells has not been investigated. Given the important role of hypoxia in driving AML progression, we hypothesized that vitexin may possess the capacity to inhibit hypoxia‐induced AML cell growth and glycolysis. Accordingly, this study was designed to validate this hypothesis and elucidate the relevant molecular mechanisms.

## 2 Materials and Methods

### 2.1 Cell Treatment

The normal human bone marrow stromal cell line HS‐5 and the human AML cell lines HL‐60 and KG‐1α (ATCC, Manassas, VA) were maintained at 37°C in RPMI‐1640 (Gibco, Grand Island, NY) supplemented with 10% FBS, 100 U/mL penicillin, and 100 μg/mL streptomycin in a humidified atmosphere containing 5% CO_2_. The cells were treated with different concentrations of vitexin (purity 99.9%; Cat. No. HY‐N0013; MedChemExpress, Monmouth Junction, NJ) at 37°C for 48 h. For hypoxia exposure, cells were cultured in an anaerobic incubator with 1% O_2_ at 37°C for 48 h.

### 2.2 Cell Viability Detection

HL‐60 and KG‐1α cells were seeded into 96‐well plates and then subjected to cell viability determination after indicated treatments. Briefly, 20 μL MTT (5 mg/mL) solution was added into each well and further incubated for 4 h at 37°C. Next, 150 μL dimethyl sulfoxide was used to exchange the medium. The absorbance values at 490 nm were measured using a spectrophotometer (USCN KIT Inc., Wuhan, China). Experiments were performed in triplicate and repeated three times.

### 2.3 The 5‐Ethynyl‐2′‐Deoxyuridine (EdU) Incorporation Assay

The proliferation of HL‐60 and KG‐1α cells was detected using the BeyoClick EdU Cell Proliferation Kit (Beyotime, Shanghai, China) following the manufacturers′ instructions. Finally, cell nuclei were stained with Hoechst 33342, provided by the kit. Images were acquired from three randomly selected fields per group using a fluorescence microscope (Olympus, Tokyo, Japan). All experiments were repeated three times.

### 2.4 Quantitative Real‐Time PCR (qRT‐PCR)

RNA was extracted from cells using Beyozol (R0011, Beyotime). Synthesis of complementary DNA was performed using a BeyoRT first‐strand cDNA Synthesis kit (D7170M; Beyotime). qRT‐PCR was performed using BeyoFast SYBR Green qPCR Mix (D7260; Beyotime) on a CFX RT‐PCR system (Bio‐Rad Laboratories, Hercules, CA). Relative mRNA expression levels were calculated with the 2^−ΔΔCt^ method. Glyceraldehyde‐3‐phosphate dehydrogenase (GAPDH) was used as an internal reference. The primers of GAPDH are: forward 5′‐CTGGGCTACACTGAGCACC‐3′ and reverse 5′‐AAGTGGTCGTTGAGGGCAATG‐3′. The primers of HK2 are: forward 5′‐GAGCCACCACTCACCCTACT‐3′ and reverse 5′‐CCAGGCATTCGGCAATGTG‐3′. The primers of PKM2 are: forward 5′‐AAGGGTGTGAACCTTCCTGG‐3′ and reverse 5′‐GCTCGACCCCAAACTTCAGA‐3′. The primers of LDHA are: forward 5′‐ATGGCAACTCTAAAGGATCAGC‐3′ and reverse 5′‐CCAACCCCAACAACTGTAATCT‐3′.

### 2.5 Western Blot Analysis

Total cellular lysates of HL‐60 and KG‐1α cells were prepared using RIPA lysis buffer (Beyotime). Protein concentrations were quantified, and 20 μg of total proteins were separated by 10% SDS‐PAGE followed by western blotting as previously described [18]. Primary antibodies (1:1000 dilution) against PCNA, Ki67, HK2, PKM2, LDHA, HIF‐1α, YAP, p‐YAP, protein kinase B (Akt), phosphorylated Akt (p‐Akt), and β‐actin were purchased from Abcam (Cambridge, MA). Horseradish peroxidase‐conjugated goat anti‐rabbit IgG H&L secondary antibody was also obtained from Abcam. Blots were developed using enhanced chemiluminescence reaction solution (Thermo Fisher Scientific, Waltham, MA). The gray value of each band was analyzed using ImageJ software (NIH, Bethesda, MD). All experiments were repeated three times.

### 2.6 Glucose Uptake and Lactate Production Detection

After the indicated treatments, the culture supernatants of HL‐60 and KG‐1α cells were collected for the measurement of glucose and lactate concentrations with a glucose assay kit and a lactate assay kit (BioVision, Mountain View, CA), respectively, according to the manufacturer′s instructions. Experiments were performed in triplicate and repeated three times.

### 2.7 Cell Transfection

For constructing HIF‐1α‐ or YAP‐depleting cells, 10 μL of HIF‐1α siRNA (10 μM, siHIF‐1α), or YAP siRNA (10 μM, siYAP), or control siRNA (10 μM, siCtrl) obtained from Santa Cruz (Santa Cruz, CA) were transfected into HL‐60 and KG‐1α cells using a Lipofectamine RNAiMAX transfection reagent (Thermo Fisher). For establishing HIF‐1α‐overexpressing cells, HL‐60 and KG‐1α cells (60,000 cells/well) were plated into 6‐well plates and then transfected with HIF‐1α‐overexpressing vector (pcDNA3.1/HIF‐1α) or control empty vector pcDNA3.1 using Attractene transfection reagent (QIAGEN, Hilden, Germany). Relative expression levels of HIF‐1α or YAP were assessed post‐transfection by western blot analysis.

### 2.8 Statistical Analysis

GraphPad Prism software (version 6.0; GraphPad Software Inc. San Diego, CA), was utilized to analyze the experimental data. The measurement data were analyzed with Student′s t‐test or one‐way analysis of variance, and results were presented as mean ± SD or SEM. The significance level was set at *p* < 0.05.

## 3 Results

### 3.1 Vitexin Inhibits Hypoxia‐Induced Increase in Growth of AML Cells

As shown in Figure 1B,C, vitexin showed a dose‐dependent inhibitory effect on the viability of HL‐60 and KG‐1α cells. However, vitexin did not affect the viability of HS‐5 cells (Figure 1D), suggesting that it is non‐cytotoxic to normal human bone marrow stromal cells. Hypoxia exposure increased the AML cell viability, while vitexin treatment dose‐dependently suppressed the hypoxia‐induced increase in cell viability (Figure 1E,F). Vitexin reduced the hypoxia‐induced increase in the percentage of EdU‐positive HL‐60 and KG‐1α cells (Figure 1G). Besides, the hypoxia‐induced increase in the expression of PCNA and Ki67 was alleviated by vitexin treatment (Figure 1H).

### 3.2 Vitexin Inhibits Hypoxia‐Induced Increase in Glycolysis of AML Cells

**FIGURE 2:**
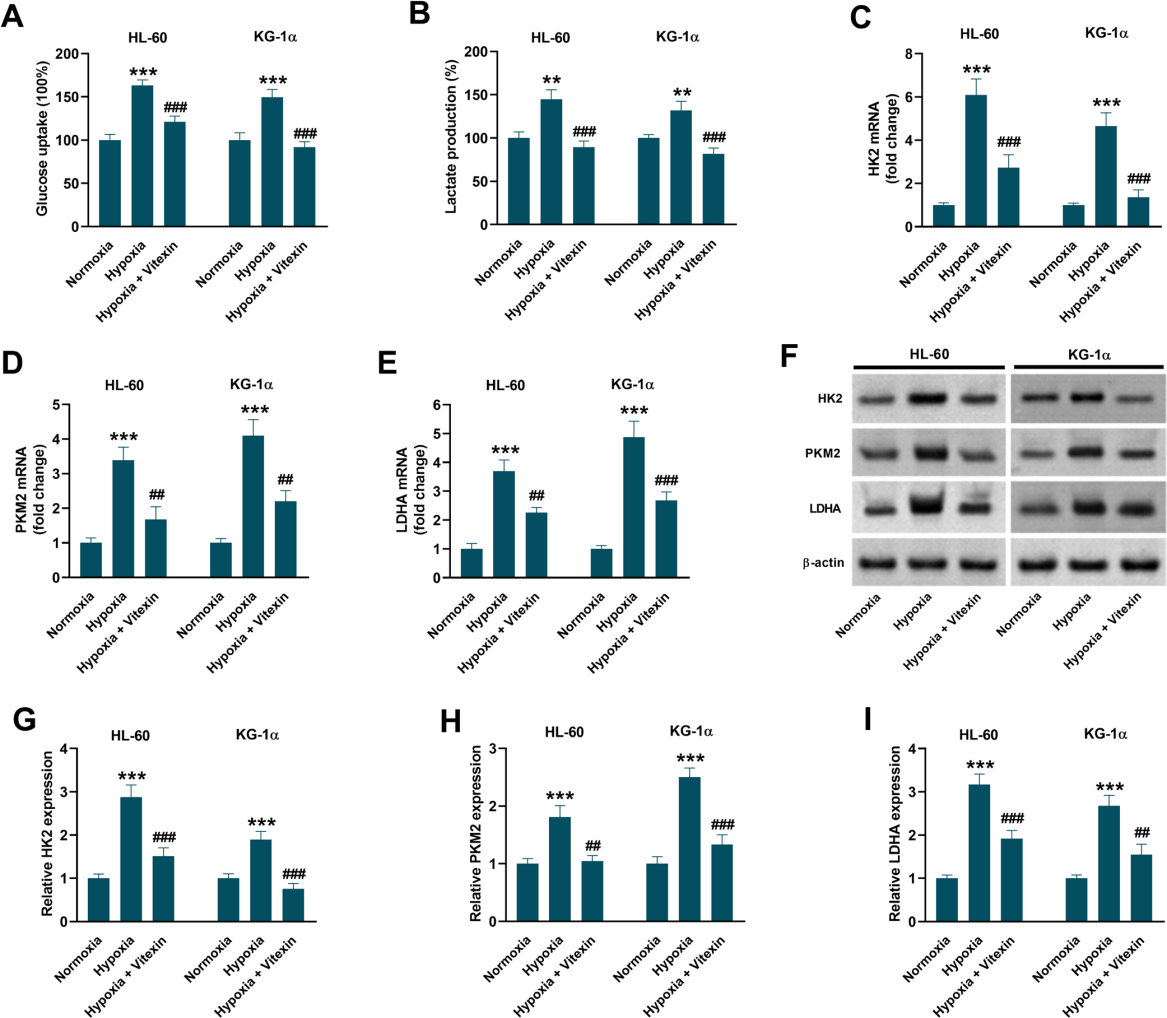
Vitexin inhibits hypoxia‐induced increase in glycolysis of HL‐60 and KG‐1α cells. HL‐60 and KG‐1α cells were treated with or without 40 μM vitexin under hypoxic conditions for 48 h. Cells under normoxic conditions served as the control group. (A) Measurement of glucose uptake. Data are presented as mean ± SEM. (B) Measurement of lactate production. Data are presented as mean ± SEM. (C‐E) qRT‐PCR for detecting the mRNA expression levels of HK2, PKM2, and LDHA. Data are presented as mean ± SD. (F‐I) Western blot analysis for detecting the protein expression levels of HK2, PKM2, and LDHA. Data are presented as mean ± SD. **p < 0.01, ***p < 0.001 vs. Normoxia group. ^##^ *p* < 0.01, ^###^ *p* < 0.001 vs. Hypoxia group.

As shown in Figure 2A,B, higher glucose uptake and lactate production were observed in the hypoxia group than in the normoxia group. Vitexin treatment reversed this hypoxia‐induced increase in glucose uptake and lactate production. The qRT‐PCR analysis showed that the mRNA expression levels of HK2 (Figure 2C), PKM2 (Figure 2D), and LDHA (Figure 2E) increased under hypoxic conditions but were inhibited by vitexin treatment. Western blot analysis showed that the protein expression levels of HK2, PKM2, and LDHA increased after hypoxia induction but decreased with vitexin treatment (Figure 2F–I).

### 3.3 Vitexin Inhibits Hypoxia‐Induced HIF‐1α Expression and the YAP Pathway Activation

**FIGURE 3:**
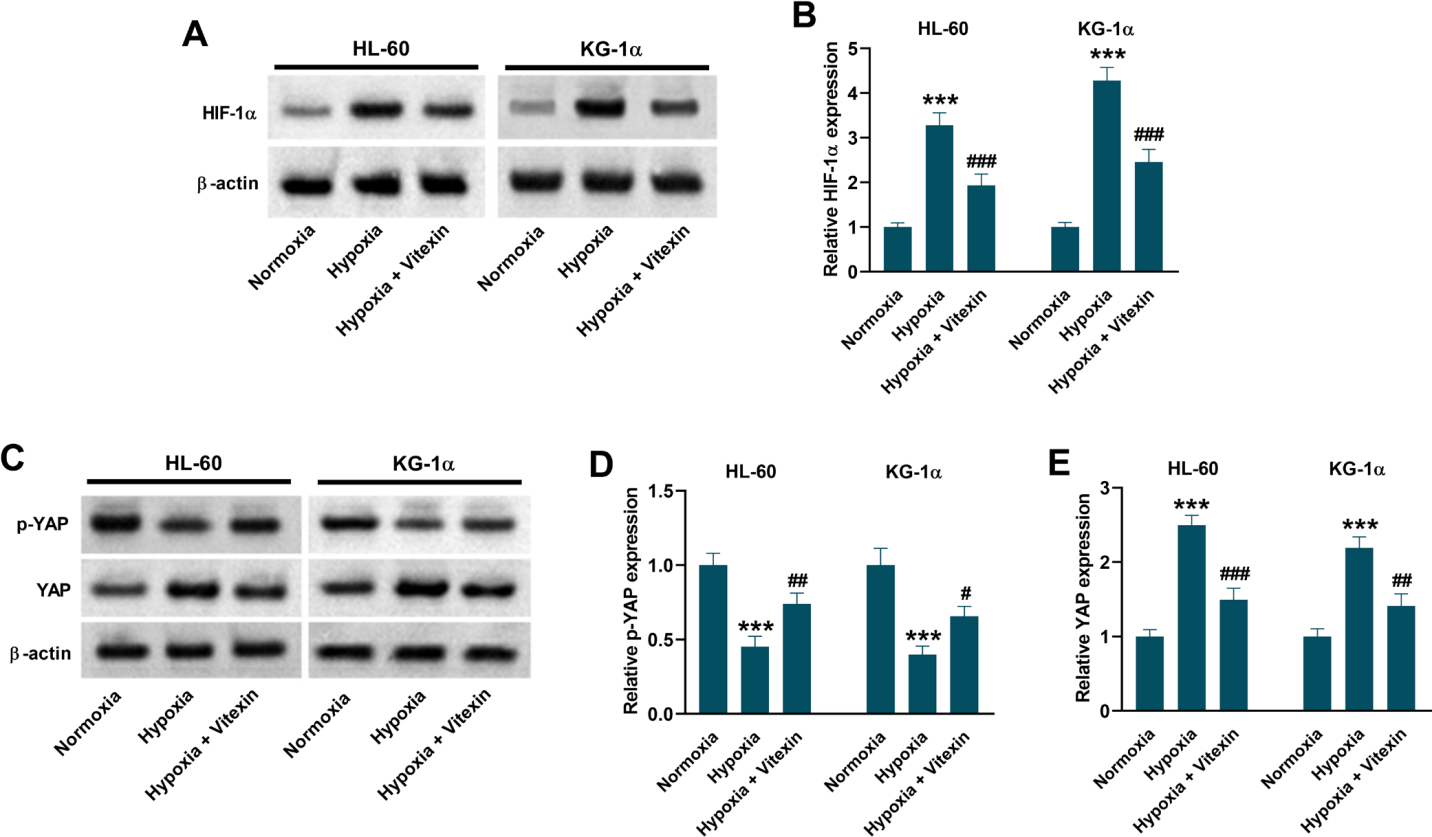
Vitexin inhibits hypoxia‐induced HIF‐1α protein expression and the YAP pathway activation in HL‐60 and KG‐1α cells. HL‐60 and KG‐1α cells were treated with or without 40 μM vitexin under hypoxic conditions for 48 h. Cells under normoxic conditions served as the control group. (A, B) Western blot analysis for detecting the expression level of HIF‐1α. (C‐E) Western blot analysis for detecting the expression levels of p‐YAP and YAP. Data are presented as mean ± SD. ***p < 0.001 vs. Normoxia group. ^##^ *p* < 0.01, ^#^ *p* < 0.05, ^###^ *p* < 0.001 vs. Hypoxia group.

To uncover the mechanisms underlying the roles of vitexin, the potential involvement of HIF‐1α and the YAP pathway was explored. A high level of HIF‐1α expression was found in hypoxia‐exposed HL‐60 and KG‐1α cells, which was prevented by vitexin treatment (Figure 3A,B). Moreover, the expression level of p‐YAP was decreased and that of YAP was increased in hypoxia‐exposed HL‐60 and KG‐1α cells, implying the activation of the YAP pathway. Hypoxia‐induced activation of the YAP pathway was reversed by vitexin treatment (Figure 3C,E).

### 3.4 The YAP Pathway Is Regulated by HIF‐1α in Hypoxia‐Exposed AML Cells

**FIGURE 4:**
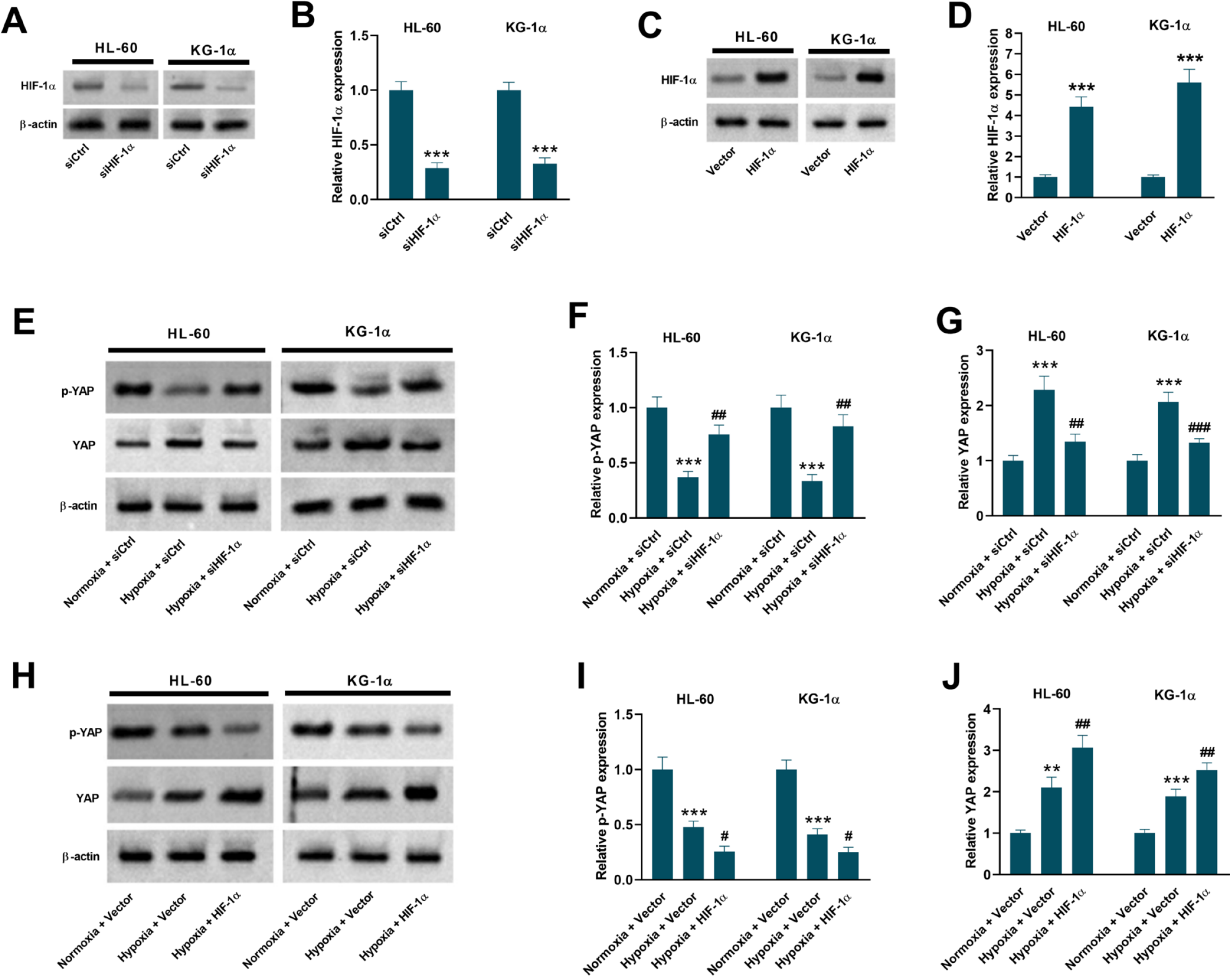
The YAP pathway is regulated by HIF‐1α in hypoxia‐exposed HL‐60 and KG‐1α cells. (A, B) HIF‐1α protein level was decreased in HL‐60 and KG‐1α cells 48 h after transfection with siHIF‐1α. ***p < 0.001 vs. siCtrl group. (C, D) HIF‐1α protein level was increased in HL‐60 and KG‐1α cells 48 h after transfection with HIF‐1α overexpression vector. ***p < 0.001 vs. Vector group. (E‐G) HL‐60 and KG‐1α cells were transfected with siCtrl or siHIF‐1α under normoxic or hypoxic conditions for 48 h. Western blot analysis was conducted to detect the expression levels of p‐YAP and YAP. ***p < 0.001 vs. Normoxia + siCtrl group. ^##^ *p* < 0.01, ^###^ *p* < 0.001 vs. Hypoxia + siCtrl group. (H‐J) HL‐60 and KG‐1α cells were transfected with control empty vector or HIF‐1α overexpression vector under normoxic or hypoxic conditions for 48 h. Western blot analysis was conducted to detect the expression levels of p‐YAP and YAP. Data are presented as mean ± SD. **p < 0.01, ***p < 0.001 vs. Normoxia + Vector group. ^#^ *p* < 0.05, ^##^ *p* < 0.01 vs. Hypoxia + Vector group. siCtrl, control siRNA. siHIF‐1α, siRNA targeting HIF‐1α. Vector, control empty vector. HIF‐1α, HIF‐1α overexpression vector.

Next, HIF‐1α‐depleting AML cells were constructed through transfection with siHIF‐1α (Figure 4A,B). HIF‐1α‐overexpressing HL‐60 and KG‐1α cells were constructed through transfection with an HIF‐1α overexpression vector (Figure 4C,D). Data in Figure 4E–G showed that hypoxia‐induced activation of the YAP pathway was blocked by siHIF‐1α, as evidenced by increased p‐YAP expression and decreased YAP expression. Additionally, overexpression of HIF‐1α enhanced the activation of the YAP pathway in hypoxia‐exposed AML cells (Figure 4H–J).

### 3.5 Roles of HIF‐1α and the YAP Pathway on the Inhibitory Effect of Vitexin on Hypoxia‐Induced AML Cell Growth

**FIGURE 5:**
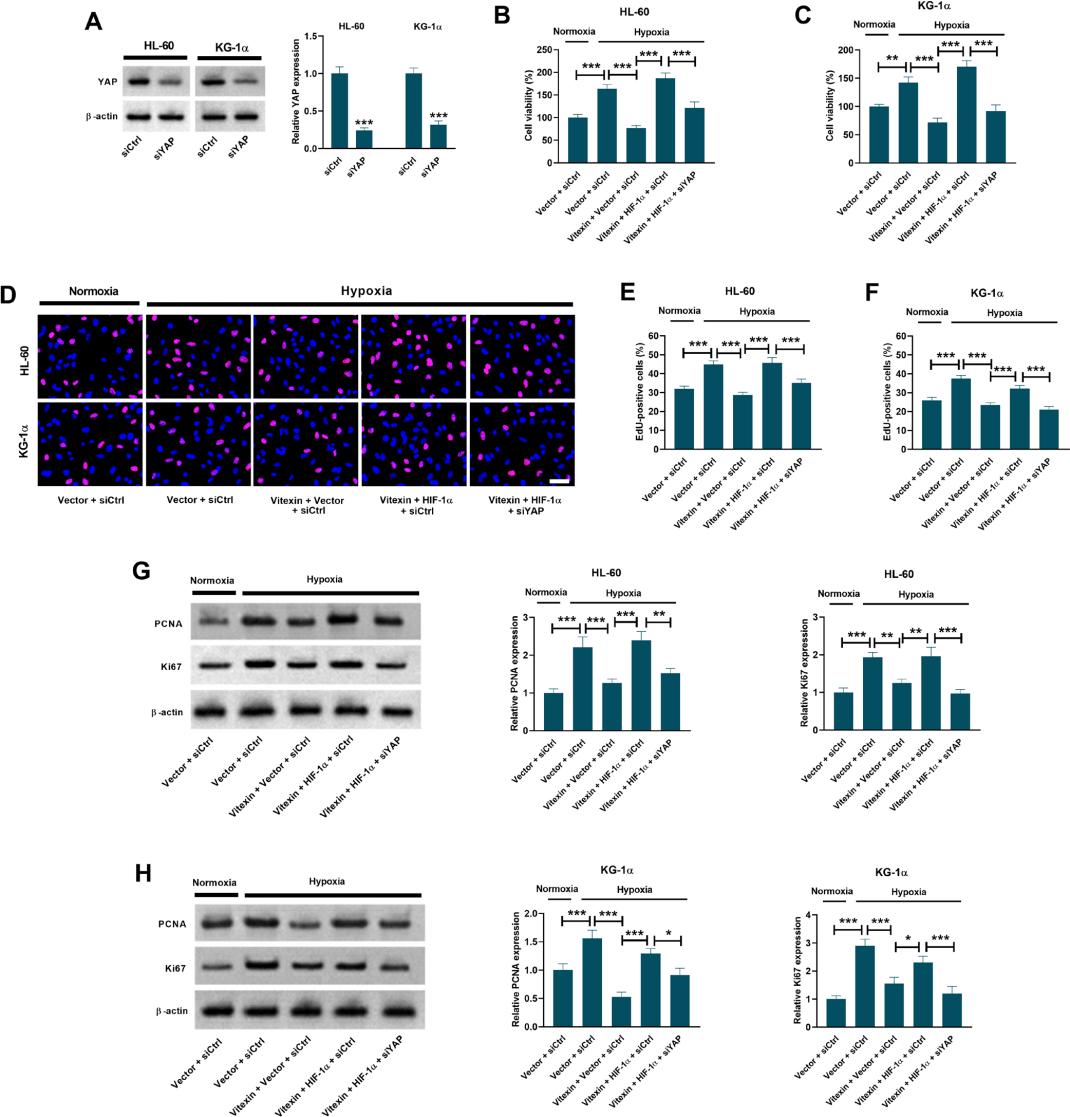
Roles of HIF‐1α or the YAP pathway in the inhibitory effect of vitexin on hypoxia‐induced AML cell growth. (A) YAP was successfully knocked down in HL‐60 and KG‐1α cells through transfection with siYAP. Data are presented as mean ± SD. ***p < 0.001 vs. siCtrl group. HL‐60 and KG‐1α cells co‐transfected with siCtrl/siYAP and Vector/HIF‐1α were treated with or without 40 μM vitexin under normoxic or hypoxic conditions for 48 h. (B, C) MTT assay was performed to assess cell viability. Data are presented as mean ± SEM. **p < 0.01, ***p < 0.001. (D‐F) EdU incorporation assay was conducted to assess cell proliferation. Scale bar = 20 μm. Data are presented as mean ± SEM. ***p < 0.001. (G, H) Western blot analysis was conducted to detect the expression levels of PCNA and Ki67. Data are presented as mean ± SD. *p < 0.05, **p < 0.01, ***p < 0.001. siCtrl, control siRNA. siYAP, siRNA targeting YAP. Vector, control empty vector. HIF‐1α, HIF‐1α overexpression vector.

Subsequently, siYAP was transfected into HL‐60 and KG‐1α cells, and YAP was successfully knocked down in the two AML cell lines (Figure 5A). Results in Figure 5B,C, showed that HIF‐1α overexpression attenuated the inhibitory effect of vitexin on the viability of hypoxia‐exposed AML cells, which was reversed by siYAP. Overexpression of HIF‐1α also attenuated the inhibitory effect of vitexin on hypoxia‐induced increases in the percentage of EdU‐positive AML cells (Figure 5D–F) and the expression levels of PCNA and Ki67 (Figure 5G,H). These effects of HIF‐1α overexpression were prevented by transfection with siYAP.

### 3.6 Roles of HIF‐1α and the YAP Pathway on the Inhibitory Effect of Vitexin on Hypoxia‐Induced Glycolysis Increase

**FIGURE 6:**
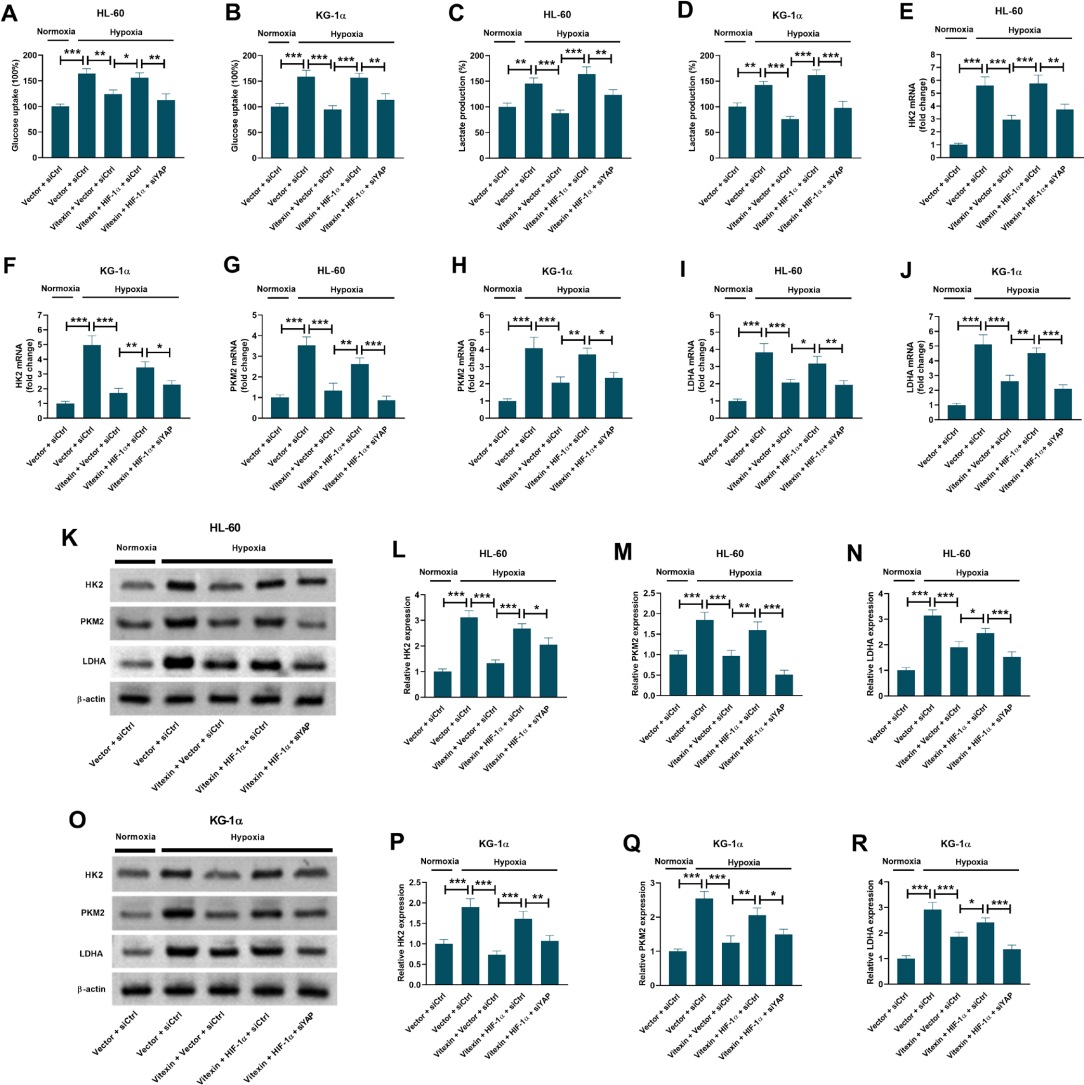
Roles of HIF‐1α or the YAP pathway in the inhibitory effect of vitexin on hypoxia‐induced glycolysis. HL‐60 and KG‐1α cells co‐transfected with siCtrl/siYAP and Vector/HIF‐1α were treated with or without 40 μM vitexin under normoxic or hypoxic conditions for 48 h. (A, B) Measurement of glucose uptake. Data are presented as mean ± SEM. (C, D) Measurement of lactate production. Data are presented as mean ± SEM. (E‐J) qRT‐PCR for detecting the mRNA expression levels of HK2 (E, F), PKM2 (G, H), and LDHA (I, J). Data are presented as mean ± SD. (K‐R) Western blot analysis for detecting the protein expression levels of HK2, PKM2, and LDHA in HL‐60 (K‐N) and KG‐1α (O‐R) cells. Data are presented as mean ± SD. *p < 0.05, **p < 0.01, ***p < 0.001. siCtrl, control siRNA. siYAP, siRNA targeting YAP. Vector, control empty vector. HIF‐1α, HIF‐1α overexpression vector.

HIF‐1α overexpression attenuated the inhibitory effects of vitexin on hypoxia‐induced increase in glucose uptake (Figure 6A,B) and lactate production (Figure 6C,D), while YAP knockdown suppressed these effects of HIF‐1α overexpression in both HL‐60 and KG‐1α cells. The inhibitory effects of vitexin on hypoxia‐induced increase in the mRNA expression levels of HK2 (Figure 6E,F), PKM2 (Figure 6G,H), and LDHA (Figure 6I,J) were partially reversed by HIF‐1α overexpression. The effects of HIF‐1α overexpression were reversed by YAP depletion. Besides, the inhibitory effects of vitexin on the hypoxia‐induced increase in the protein expression levels of HK2, PKM2, and LDHA were reversed by HIF‐1α overexpression. Furthermore, these effects of HIF‐1α overexpression were attenuated by YAP depletion in both HL‐60 (Figure 6K–N) and KG‐1α cells (Figure 6O–R).

## 4 Discussion

Vitexin cooperates with hyperbaric oxygen and enhances the radiosensitivity of gliomas, which is correlated with the inhibition of HIF‐1α protein expression and HIF‐1α signaling downstream proteins [19]. Vitexin enhances the radiosensitivity of glioma in a mouse subcutaneous xenograft glioma model by regulating the miR‐17‐5p/miR‐130b‐3p/PTEN/HIF‐1α pathway [20]. Vitexin inhibits the malignant progression of gastric cancer by suppressing growth and metastasis via inactivation of the PI3K/AKT/mTOR/HIF‐1α signaling pathway [21]. Chio et al. demonstrated that vitexin has anti‐metastatic potential in rat pheochromocytoma cells by inhibiting hypoxia‐induced activation of the c‐Jun N‐terminal kinase pathway [22]. These results suggest that therapeutic strategies using vitexin with the underlying mechanisms involving hypoxia and HIF‐1α signaling are a major focus area in cancer research.

Hypoxia is a pivotal component of the bone marrow microenvironment and hematopoietic stem cell niche, and exerts a critical influence on the pathogenesis of AML and the response to therapeutic interventions [23]. The bone marrow microenvironment is characteristically maintained in a hypoxic state under physiological conditions, where low oxygen partial pressure contributes to the preservation of hematopoietic homeostasis. Traditionally, hypoxia has been recognized as a key niche feature that supports the quiescence of normal hematopoietic stem cells [24]. In AML, however, the malignant expansion of leukemic stem cells exacerbates bone marrow hypoxia [25]. In contrast to its physiological role in supporting normal hematopoietic stem cells, the hypoxic bone marrow microenvironment in AML evolves into a “malignant niche” that actively promotes leukemic stem cell survival and proliferation [26]. As a significant transcription factor related to hypoxia induction, HIF‐1α triggers the induction of a wide range of genes that are responsible for regulating metabolism, angiogenesis, apoptosis, and the cell cycle [27]. It has been reported that HIF‐1α is overexpressed in patients with AML compared to normal controls [28]. Moreover, overexpression of HIF‐1α is associated with poor prognosis and contributes to chemoresistance in AML [29, 30]. It has been proven that therapies targeting hypoxia and HIF‐1α are effective in the treatment of mouse models [31]. The hypoxia‐activated prodrug PR104 targeting hypoxia in refractory/relapsed AML and acute lymphoblastic leukemia is currently undergoing clinical trials [32]. Furthermore, inhibition of HIF‐1α by CdCl_2_ (an inhibitor of HIF‐1α) or siHIF‐1α reduces AML cell viability under hypoxic conditions [33, 34].

Under normoxic conditions, HIF‐1α keeps at a low or an undetectable level, since it is hydroxylated by the prolyl hydroxylase‐domain proteins (PHDs), ubiquitinated by the binding of the von Hippel–Lindau protein, and finally degraded by the 26S proteasome; however, under hypoxic conditions, the activities of PHDs are reduced, leading to decreased hydroxylation of HIF‐1α and subsequent accumulation of HIF‐1α in cells [35]. The stability of HIF‐1α is also regulated by the phosphatidylinositol 3 kinase (PI3K)/protein kinase B (Akt) pathway under hypoxic conditions [36]. We found that treatment with vitexin or LY294002 (an inhibitor of the PI3K/Akt pathway) suppressed the expression of HIF‐1α and p‐Akt in AML cells under hypoxic conditions (Figure S1). Under normoxic conditions, however, vitexin or LY294002 treatment decreased p‐Akt levels without affecting HIF‐1α expression (Figure S1). These results led us to deduce that vitexin suppressed the level of HIF‐1α by inactivating the PI3K/Akt pathway under hypoxic conditions. Notably, under normoxic conditions, HIF‐1α is inherently maintained at an extremely low basal level, which limits the possibility of further reducing its expression via inhibition of the PI3K/Akt pathway. This observation is consistent with previous studies showing that LY294002 treatment reduces the level of HIF‐1α in thyroid carcinoma and hepatocellular carcinoma cells under hypoxic conditions but fails to exert this effect under normoxic conditions [37, 38]. Nevertheless, siRNA‐mediated knockdown of HIF‐1α still reduces the expression level of HIF‐1α and inhibits the viability of AML cells under normoxic conditions [39].

**FIGURE 7:**
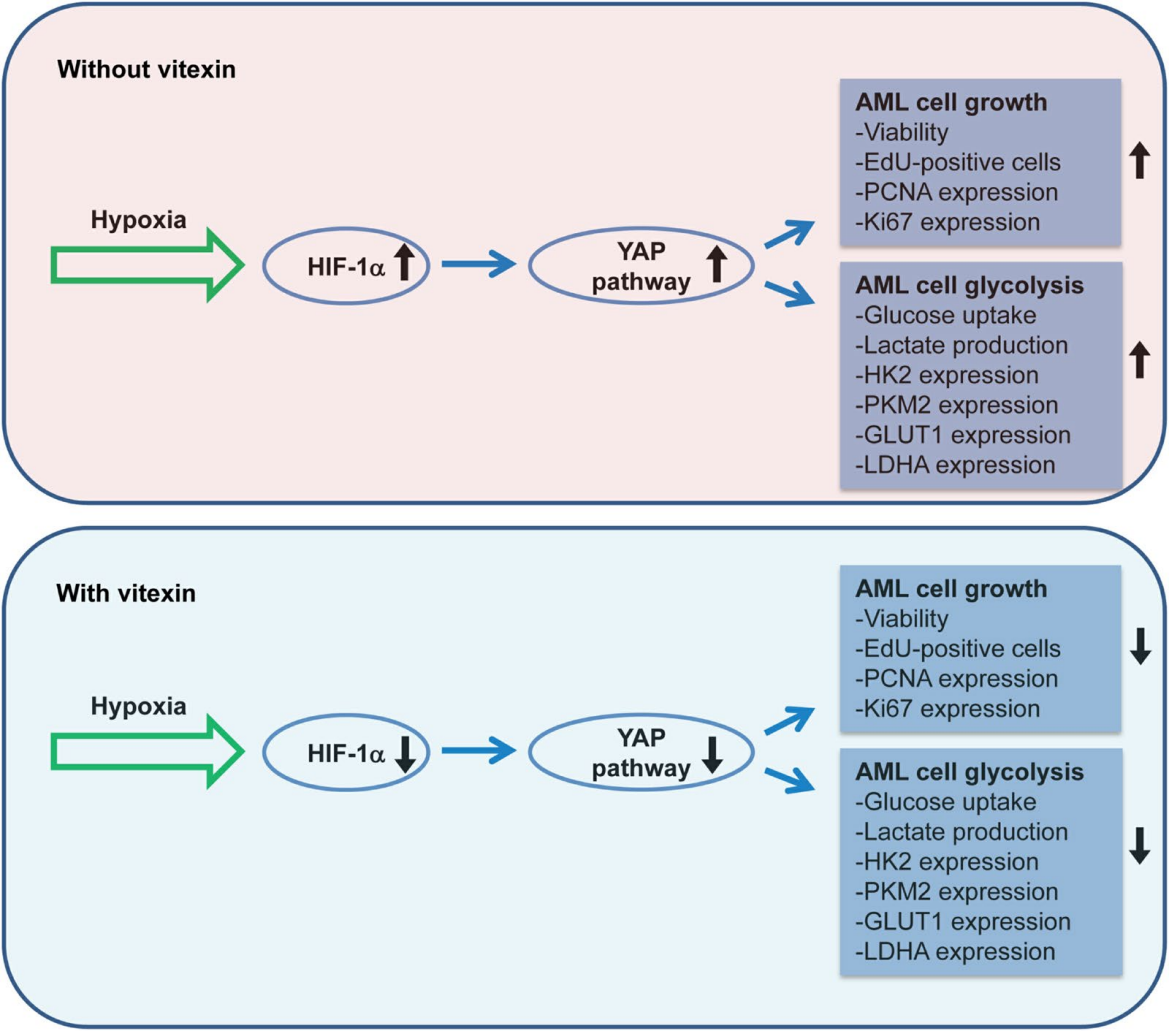
Schematic overview of the mechanisms underlying the antitumor roles of vitexin in AML cells. Under hypoxic conditions, HIF‐1α expression was elevated, which induced the activation of YAP pathway and subsequent AML cell growth and glycolysis. Vitexin treatment prevented hypoxia‐induced growth and glycolysis of AML cells by suppression of HIF‐1α‐modulated YAP pathway.

YAP is a core transcriptional coactivator that plays a vital role in the regulation of stem cell maintenance, proliferation, apoptosis, and tissue homeostasis [40]. In addition, previous studies have shown that YAP is regulated by HIF‐1α in cervical cancer and AML cells under normoxic conditions [13, 33]. In hepatocellular carcinoma cells, YAP binds to HIF‐1α and sustains the stability of HIF‐1α protein under hypoxic stress, thereby promoting cell glycolysis [12]. Another study shows that HIF‐1α forms a regulatory loop with YAP, enhances HIF‐1α stability, and elevates HIF‐1α activity, thus coordinating hypoxia‐induced adriamycin resistance in AML cells [33]. Consistent with these findings, our results showed that the YAP pathway was regulated by HIF‐1α in hypoxia‐exposed AML cells. Moreover, HIF‐1α/YAP pathway mediated the inhibitory effects of vitexin on hypoxia‐induced AML cell growth and glycolysis (Figure 7). However, the current study has several limitations. First, it is uncertain whether vitexin plays an inhibitory role in the growth and glycolysis of AML cells in vivo, which warrants further studies using nude mouse models. Second, the Hippo pathway has been reported to affect YAP expression; however, it was unclear whether the Hippo pathway was involved in the HIF‐1α/YAP axis in this study. Therefore, the mechanism underlying the action of vitexin needs to be explored.

Our findings revealed a novel mechanism by which vitexin inhibits AML progression. Vitexin prevented hypoxia‐induced increase in growth and glycolysis in AML cells by regulating HIF‐1α expression. Moreover, YAP served as a downstream effector of HIF‐1α. These results are helpful in understanding the mechanisms of action of vitexin in AML.

## Conflicts of Interest

The authors declare no conflicts of interest.

## Supporting information


**Figure S1:** Effect of vitexin on HIF‐1α expression and the PI3K/Akt pathway in AML cells under normoxic or hypoxic conditions. (A‐F) HL‐60 and KG‐1α cells were treated with 40 μM vitexin or 10 μM LY294002 (an inhibitor of the PI3K/Akt pathway) under normoxic or hypoxic conditions for 48 h. Western blot analysis was conducted to detect the expression levels of HIF‐1α, p‐Akt, and Akt. Data are presented in the form of mean ± SD. ns, no statistical significance. *p < 0.05, **p < 0.01, ***p < 0.001.

## Data Availability

The data that support the findings of this study are available from the corresponding author upon reasonable request.
