# Supplementary material for: Vitexin Attenuates the Growth and Glycolysis of Acute Myeloid Leukemia Cells by Suppressing the HIF‐1α‐Modulated YAP Pathway Under Hypoxic Conditions
Source: Kaohsiung J Med Sci. 2025 Sep 25;42(3):e70111. doi: 10.1002/kjm2.70111 (PMC12955921; doi:10.1002/kjm2.70111)
Supplement: Supplementary file 1 — Figure S1: Effect of vitexin on HIF‐1α expression and the PI3K/Akt pathway in AML cells under normoxic or hypoxic conditions. (A‐F) HL‐60 and KG‐1α cells were treated with 40 μM vitexin or 10 μM LY294002 (an inhibitor of the PI3K/Akt pathway) under normoxic or hypoxic conditions for 48 h. Western blot analysis was conducted to detect the expression levels of HIF‐1α, p‐Akt, and Akt. Data are presented in the form of mean ± SD. ns, no statistical significance. *p < 0.05, **p < 0.01, ***p < 0.001. [file KJM2-42-e70111-s001.docx]

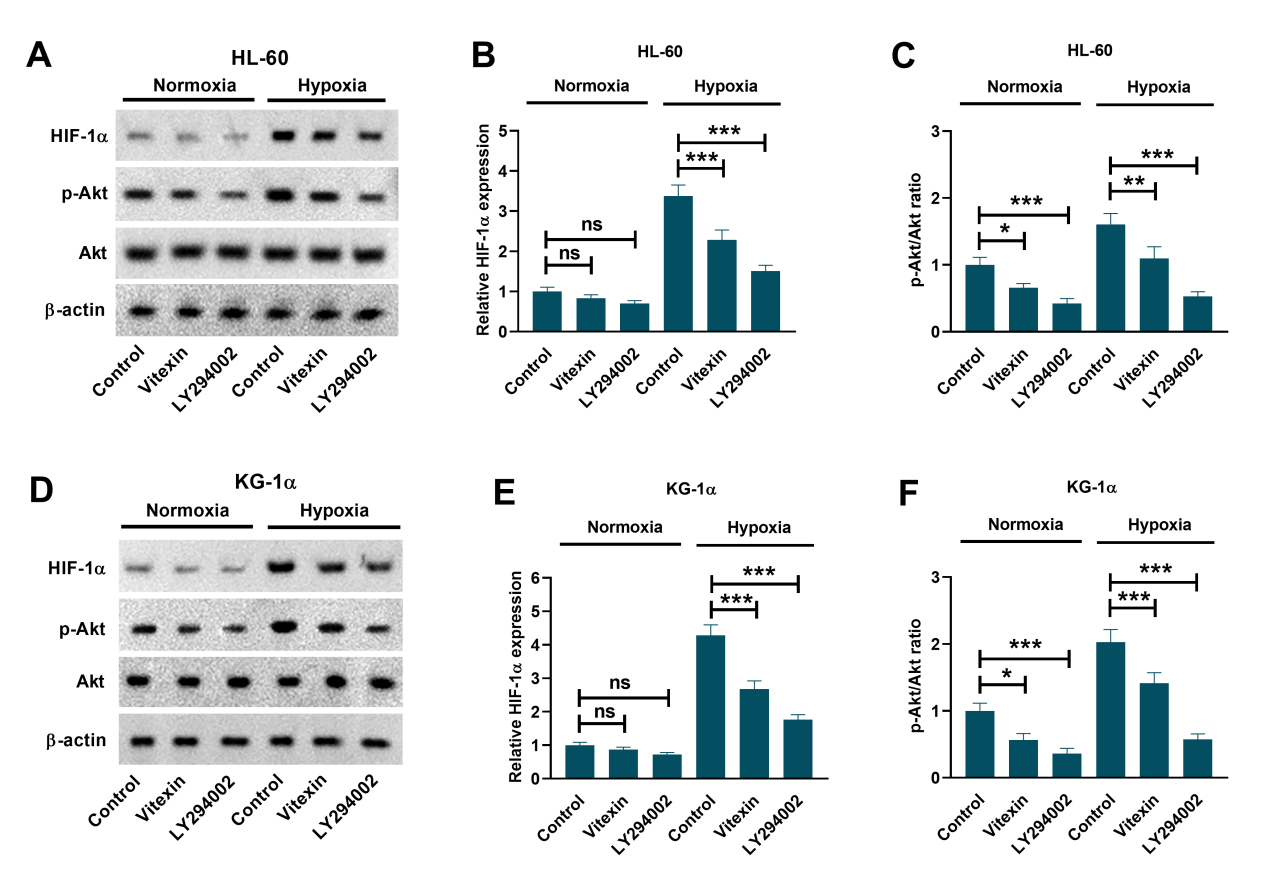


**Supplementary Figures S1.** Effect of vitexin on HIF-1α expression and the PI3K/Akt pathway in AML cells under normoxic or hypoxic conditions. (A-F) HL-60 and KG-1α cells were treated with 40 μM vitexin or 10 μM LY294002 (an inhibitor of the PI3K/Akt pathway) under normoxic or hypoxic conditions for 48 h. Western blot analysis was conducted to detect the expression levels of HIF-1α, p-Akt, and Akt. Data are presented in the form of mean ± SD. ns, no statistical significance. **p* < 0.05, ***p* < 0.01, ****p* < 0.001.
